# Chiral clues to lipid identity

**DOI:** 10.1016/j.jlr.2024.100710

**Published:** 2024-11-20

**Authors:** Ujjalkumar S. Das, Garret A. FitzGerald

**Affiliations:** Institute for Translational Medicine and Therapeutics, Perelman School of Medicine, University of Pennsylvania, Philadelphia, PA, USA

Oxylipins include octadecanoids, eicosanoids, and docosadecanoids, named for the respective lengths (18, 20, and 22) of their carbon chains. Despite them being evanescent and of low abundance in biological fluids, they exhibit remarkably diverse biological activity which continues to be revealed (1, 2). These pathways have yielded a remarkable number of drug targets, despite meager support from human genetics, including aspirin and other non-steroidal anti-inflammatory drugs (NSAIDs), leukotriene receptor antagonists, and a series of prostanoid receptor (Pr) agonists and antagonists in development. Besides enzymatically formed oxylipins, such as the eicosanoids, free radical catalyzed isomers—iso-eicosanoids—have proven utility as indices of lipid peroxidation (3). Oxylipins may also have contrasting biological activities, often revealed first *in vivo* by receptor deletions. Examples include the effects of prostacyclin (PGI_2_) versus thromboxane (TxA_2_) on platelet function and vascular tone (4) or PGE_2_ promoting or restraining inflammation by differentially activating its 4 EPrs (5).

Recently, controversy has surrounded the biological importance of a group of oxylipins termed Specialized Pro-Resolving Mediators (SPMs), suggested as endogenous mediators of the resolution of inflammation and as leads for synthetic molecules of therapeutic potential (6). Specifically, questions have been raised about the plausibility of their biosynthetic pathways *in vivo* (7, 8), their putative receptors (9), the methodology deployed to measure them (10) and whether they exist in concentrations sufficient to exert biological activity (11). Although failing to support SPMs as cognate ligands for their proposed receptors Alnouri *et al.* (9) have recently shown that the SPM, Resolvin (Rv)D5, can function as a biased positive modulator of the EPr4 in macrophages to alter its G protein coupling, switching the PGE_2_ response to increase phagocytosis, consistent with resolution of inflammation, *in-vitro*. Indeed, when RvD5 was infused into mice treated with zymosan to induce inflammation, it depressed peritoneal accumulation of inflammatory leucocytes in an EPr4 dependent manner. However, the concentrations of RvD5 used in the macrophage studies (30 nM) and measured in plasma (3,152–5,961 pg/ml) at rated of infusion to achieve such pro-resolving properties exceed by orders of magnitude reported endogenous concentrations using any credible methodology.

Given this controversy and the potential for putative SPMs to be autooxidation products (12), there is particular interest in determining the enantioselective formation of oxylipins, especially putative SPMs. Chiral oxylipins include regioisomers or positional isomers (the same formula with a different structure) stereoisomers (the same formula and structure with a different spatial conformation) composed of geometrical isomers (*cis-/trans-)* and optical isomers (*R-* and *S-*enantiomers, one chiral center with opposite configuration; diastereomers, more than one chiral centers with opposite configuration). By contrast, free radical-catalyzed products, such as iso-eicosanoids, have no stereoselectivity (13).

In this issue of *Journal of Lipid Research*, Nils Schebb’s group reports a novel approach to chiral analysis of oxylipins, Multiple Heart Cutting (MHC) achiral-chiral 2D-LC-MS/MS (14). They perform absolute quantification of oxylipin diversity in a first dimension (^1^D) along with enantioselective oxylipin analysis in a second (^2^D) orthogonal dimension, allowing the rapid calculation of the enantiomeric fraction and quantification of the enantiomeric isomers in a sample (Fig. 1).

**Fig. 1:**
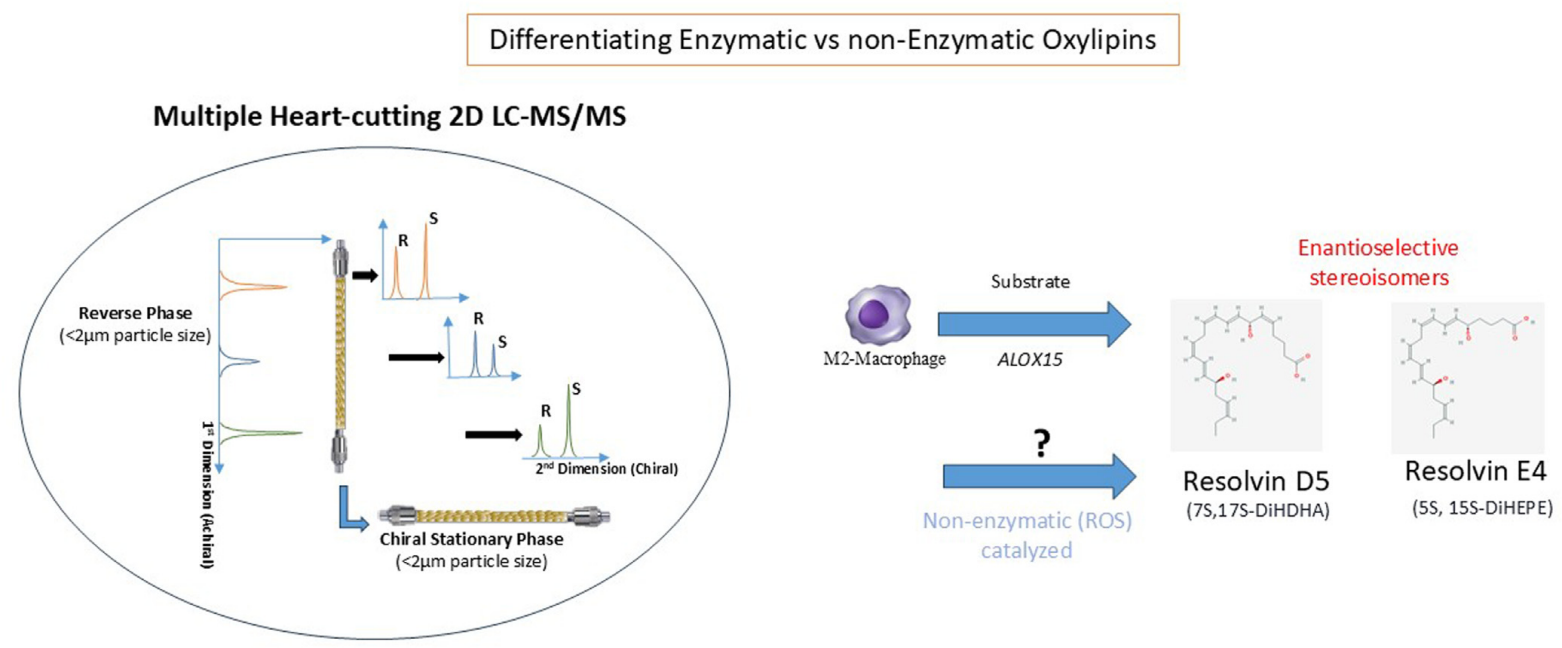
Depiction of multiple heart cutting (MHC) achiral-chiral 2D-LC-MS/MS supporting ALOX15 enzymatic production of RvD5 and RvE4 in vitro while separating non-enzymatic contribution by reactive oxygen species (ROS).

A challenge to the analysis of oxylipins using LC-MS/MS is the presence of structural/stereo-isomeric molecules that have isobaric masses with similar fragmentation patterns and elution time profiles. This has been addressed by standard reverse phase (RP) ultra-high performance liquid chromatography (UHPLC). Here, using an RP C18 column for ^1^D achiral separation, the authors achieved narrow peaks with full peak width at half maximum (FWHM) of 4–5 s with sufficient resolution among isobars and positionally isomeric oxylipins. The selected sections/peaks were then transferred to ^2^D (chiral column), where the run time was a bit longer (32 min vs. ∼25 min) than RP UHPLC based methods. As expected ^1^D achiral method was unable to separate enzymatically formed dihydroxy-fatty acid diastereomers - (5(*R/S*),15(*S*)-DiHETE, 5(*R/S*)-,15(*S*)-DiHEPE and 7(*R/S*),17(*S*)-DiHDHA).

Specialized chiral stationary phases (CSPs), such as tris(3,5-dimethylphenylcarbamate) derivatives of amylose, for example, the sub-2 μm particle size amylose column (Chiralpak IA-U column 100 × 3.0 mm, 1.6 μm) using RP settings have been increasingly favored (14). Progress in the field included the development of chiral LC/electron capture atmospheric pressure chemical ionization (ECAPCI)-MS for analysis of endogenous HETE, HODE and EET enantiomers as their pentafluorobenzyl (PFB) ester derivatives (15). Normal phase (NP) chromatography coupled with ECAPCI-MS on a Chiralpak AD-H column is sensitive compared to conventional APCI (16) but under RP settings, sensitivity for detection of the PFB derivatives was inferior to standard ESI-based MS methods (17). Additional refinements included the quantification of 16 TriHOME isomers in a single lengthy (107 min) run (18) and the use of ^1^D chiral-SFC to resolve regio- and stereoisomers (epoxides and diols) and compounds with multiple chiral centers (Triols) in a single chromatographic run and allowed quantification of 103 chiral octadecanoids (19).

Schebb’s MHC achiral-chiral 2D LC-MS/MS approach combines a highly efficient sub-2 μm RP UHPLC method with sub-2 μm amylose based CSP enabled enantioselective chiral chromatography, providing enhanced sensitivity, selectivity, and stereospecific (3S) analysis of chiral oxylipins compared to published methods. This is made possible by MHC of the resolved peaks of the ^1^D RP column onto the ^2^D chiral column (Chiralpak IA-U column 50 × 3.0 mm, ie amylose tris(3,5-dimethylphenylcarbamate) material immobilized on 1.6 μm silica (Daicel) for enantioselective separation. The ^2^D chiral separation is achieved within 1.8 min/heart cut for up to 20 oxylipins enantiomers with a FWHM of < 2.5 s, a marked improvement on traditional enantioselective analysis (20). However, 2 injections were required to cover 45 oxylipins enantiomers and the run time for the two dimensions extends to 45 min introducing considerations of cost and chemical stability when running many biological samples.

The lowest limit of quantification (LLOQ) was calculated using signal-to-noise (S/N) ≥5 times the baseline. Using ^1^D achiral chromatography, the LLOQ ranged from 0.04 to 7.5 pg on the column for the 45 enantiomeric pairs comparable to published reports. Achieving a similar LLOQ on 2D chromatography may reflect a reduction in matrix effects posed by the endogenous compounds due to heart cutting and the minimal sample transfer to the MS for analysis. Interestingly, Cebo *et al.* (14) reported high resolution of HETE, HODE, HEPE, and HDHA enantiomeric pairs, ranging from 1.81 to 14.97 using only a ^1^D enantioselective method. These selected chiral oxylipins exhibited lower resolution, ranging from 0.74 to 8.30, with Schebb’s method, probably due to the short column length and fast separation at a high flow rate (0.9 ml/min) on the ^2^D chiral stationary phase.

Importantly, the authors then demonstrated clear separation of all four possible stereoisomers of dihydroxy-FA (5,15-DiHEPE, 5,15-DiHETE, and 7,17-DiHDHA) in oxidized plasma and by human M2 macrophages confirmed with authentic reference (enantiopure 5(*S)*,15(*S)*-DiHETE, 5(*S)*,15(*S)*-DiHEPE and 7(*S)*,17(*S)*-DiHDHA) and enzymatically generated standards (5(*R/S*),15(*S*)-DiHETE, 5(*R/S*),15(*S*)-DiHEPE and 7(*R/S*),17(*S*)- DiHDHA) generated from their precursors ((*R,S*)-HETE, 5(*R,S*)-HEPE and 7(*R,S*)-HDHA) by human 15-LOX. The appearance of racemates in oxidized plasma reflects ROS-mediated generation of stereoisomers. These could now be clearly separated from enzymatic products leading to correct relative quantification of each isomer. Interestingly and of relevance to the study of Alnouri *et al.* (9), the capacity of human M2 macrophages to form 5(S),15(S)-DiHEPE (RvE4) and 7(S),17(S)-DiHDHA (RvD5) as enzymatic products could be confirmed.

## Conclusion

The interest in free radical catalyzed isomers as quantitative indices of oxidative stress in vivo, the susceptibility of biological samples to peroxidation during storage, the need to determine whether putative SPMs are merely autooxidation products and if not, to interrogate their biological importance all highlight the need for enantiospecific analysis (21). Further provision of standards, refinement of CSPs, and combinatorial technical advances such as that by Shebb and colleagues will advance our quest for sensitive, specific, and rapid stereospecific approaches to the analysis of oxylipins.

### Data availability

All supporting data are provided within the manuscript, supplementary data and supplementary tables.

## Conflict of interest

The authors declare that they have no conflicts of interest relevant to the contents of this article.
